# Radio-iodine refractory thyroid cancer patients: a tailored follow-up based on clinicopathological features

**DOI:** 10.1007/s40618-023-02076-6

**Published:** 2023-04-21

**Authors:** L. Lorusso, E. Minaldi, G. Esposito, P. Piaggi, V. Bottici, S. Brogioni, C. Giani, L. Valerio, E. Molinaro, R. Elisei, L. Agate

**Affiliations:** Endocrinology Unit, Department of Clinical and Experimental Medicine, University of Pisa, Via Paradisa 2, 56124 Pisa, Italy

**Keywords:** Radioiodine-refractory thyroid cancer, Tyrosine kinase inhibitors, Metastatic differentiated thyroid cancer, Systemic therapy in thyroid cancer

## Abstract

**Objective:**

To report the experience of a single center for the selection of radioiodine-refractory (RAIR) thyroid cancer patients (RAIR-TC) who needed tyrosine kinase inhibitor (TKIs) treatment.

**Patients and methods:**

We evaluated all features of 279 RAIR-TC patients both at the time of diagnosis and at the RAIR diagnosis.

**Results:**

Ninety-nine patients received indication to TKIs (Group A), while 180 remained under active surveillance (Group B). Group A had greater tumor size, more aggressive histotype, more frequent macroscopic extrathyroidal extension, distant metastases, advanced AJCC stage, and higher ATA risk of recurrence. After RAIR diagnosis, 93.9% of Group A had progression of disease (PD) after which TKIs’ therapy was started. The remaining 6.1% of patients had a so severe disease at the time of RAIR diagnosis that TKIs’ therapy was immediately started. Among Group B, 42.7% had up to 5 PD, but the majority underwent local treatments. The mean time from RAIR diagnosis to the first PD was shorter in Group A, and the evidence of PD within 25 months from RAIR diagnosis was associated with the decision to start TKIs.

**Conclusions:**

According to our results, a more tailored follow-up should be applied to RAIR-TC patients. A too strict monitoring and too many imaging evaluations might be avoided in those with less-aggressive features and low rate of progression. Conversely, RAIR-TC with an advanced stage at diagnosis and a first PD occurring within 25 months from RAIR diagnosis would require a more stringent follow-up to avoid a late start of TKIs.

## Introduction

Differentiated thyroid carcinoma (DTC) has a high overall survival, reaching 98.4% at 5 years (SEER) [1] and up to 96.4% at 35 years [2]. Local recurrence in the thyroid bed or metastatic cervical lymph nodes occurs in approximately 20% of patients, while distant metastases are present in approximately 10% of cases (lungs in 50% followed by bone in 25%). In cases of distant metastases, the survival at 5 years drops to 53.3% [3]. About 50% of metastases are present at diagnosis, while in the other patients, they are found after a median follow-up of 3–4 years [4]. If the distant metastases maintain their capability to take up radioiodine (RAI), the prognosis of patients with distant metastases remains more favorable. Nevertheless, 5–15% of DTC and 50% of metastatic DTC will become refractory to RAI (RAIR) with a significant decrease in the overall survival rate (less than 10% at 10 years) [4]*.*

The increasing knowledge of the mechanisms underlying thyroid cancer (TC) tumorigenesis, involving tyrosine kinase receptors and tyrosine kinase in general, has led to the development of tyrosine kinase inhibitor drugs (TKIs) that are specific small oral molecules able to inhibit multiple kinases [5].

To date, three different TKIs have been approved for the treatment of patients with advanced RAIR-TC and poorly differentiated thyroid carcinoma (PDTC): sorafenib and lenvatinib that can be used as first or second line, and cabozantinib recently approved as second line. Although no improvements in overall survival were reported, probably due to the expected crossover to the drug for those patients on placebo with documented progressive disease (PD) in phase 3 trials, all the three mentioned above TKIs showed a significant improvement in progression-free survival (PFS) compared to placebo [6–9].

Although the DECISION, the SELECT, and the COSMIC-311 studies [6–8] provided much information about the efficacy of sorafenib, lenvatinib, and cabozantinib, respectively, their inclusion criteria were very stringent, particularly when considering PD according to RECIST and health status. These criteria are not always respected in clinical practice, and in the real world, the criteria for the time of starting therapy and the selection of patients to be treated are still an unmet need.

With the present study, we share the experience of a single referral tertiary center for the management of thyroid cancer, describing the selection of RAIR-TC patients to initiate systemic therapy, and evaluating, at the same time, the clinical and pathological features of a control group of “untreated” RAIR-TC patients. In particular, our attention was focused on the clinical and pathological features of RAIR-TC patients not only at the time of diagnosis but also at the time of RAIR diagnosis. The final aim of the study was to distinguish those cases who require to be followed up more strictly from those who can be submitted to less-frequent clinical and imaging controls.

## Patients and methods

We retrospectively evaluated the epidemiological, clinical, and pathological data of 279 consecutive patients [136 men (48.7%) and 143 women (51.3%)] with differentiated and poorly differentiated RAIR-TC, referred to the Endocrine Unit of the University Hospital of Pisa between June 2016 and December 2019 to undergo restaging of their neoplastic disease with biochemical and radiological procedures. The mean follow-up time of the study group from the initial diagnosis to the last visit or death or to the beginning of TKIs’ therapy was 10.5 ± 6.7 years (median 9.4 years; range 0.2–36.4 years).

All patients underwent total thyroidectomy and received at least one RAI treatment (131-I). One hundred and fifteen of 279 (41%) patients also underwent neck lymph-node dissection.

The mean number of RAI treatments was 3 ± 1.8, and the mean cumulative administered activity of 131-I was 390.3 ± 283.2 mCi (median 296 mCi; range 30–1748) in hypothyroidism or after receiving recombinant human TSH (Thyrogen, Genzyme).

Since no cured disease was obtained after the initial treatments, all patients received therapy with levothyroxine using a suppressive dose regimen (TSH ≤ 0.1).

Tissue specimens were classified as papillary thyroid cancer (PTC), follicular thyroid cancer (FTC), including Hürthle cells tumors (HCTC), or PDTC according to WHO standards. When the first surgery was performed in another center (66%), the histological diagnosis was confirmed by a pathologist at the University Hospital of Pisa with a new examination of the histological slides. TNM classification and AJCC staging have been revised according to the 8th edition of the AJCC/TNM staging system of thyroid cancer [11]. To estimate the risk of recurrent/persistent disease, all patients were classified according to the initial risk stratification system proposed by the 2015 American Thyroid Association guidelines [12] (ATA risk) into three categories: low, intermediate, and high risk of recurrence. Patients diagnosed with PDTC were all considered at high risk of recurrence.

After the initial treatments, all patients received a regular follow-up (every 3, 6, or 12 months) with neck ultrasound, clinical examination, and laboratory assessment [thyroglobulin (Tg) and Tg- autoantibody (TgAb) measurements]. Since PDTC were also included in the study, Tg trend and AbTg trend were not used as predictive factors of needing to start TKIs.

A computed tomography (CT) scan was performed after 131-I treatment when high levels of serum Tg were present in association with a negative whole-body scan (WBS) or when WBS showed distant metastases and regardless the result of neck ultrasound. Other imaging procedures (nuclear magnetic resonance, 18-FDG-positron emission tomography, and bone scintigraphy) were used whenever necessary. A CT scan with contrast medium was also used for the restaging of disease. In patients with known distant metastases, CT scans were performed every 6–12 months based on Tg/AbTg changing trends and the location of the metastases. The presence of disease progression was defined using RECIST 1.1 [13]. RAIR was defined according to the ATA guideline criteria [12].

The beginning of TKIs’ therapy was decided by a multidisciplinary team when, according to the current ATA and ETA guidelines, at least one of the following criteria was fulfilled: (1) evidence of PD, according to RECIST, in multiple locations and/or organs; (2) evidence of a metastatic disease associated with a high risk of imminent death or morbidity or mortality within 6 months (i.e., lung or node metastasis rapidly invading the respiratory tract), independently from the evidence of PD; (3) symptomatic disease (i.e., dyspnea and pain) unsuitable for local treatments. Conversely, TKIs therapy was not initiated when there was no evidence of PD or, if present, it was due to (a) the development of one or a few new or increasing lesions that were possible to treat with local treatments; (b) the increase in some asymptomatic small metastases that may be kept under follow-up; and (c) the development of new small lesions (< 1 cm) with no clinical significance.

### Statistical analysis

Categorical variables are expressed as counts and percentages, and continuous variables are expressed as medians and ranges (min–max) because of a skewed distribution. Chi-square or Fisher’s exact tests were used to evaluate the association between categorical variables. Normality of the data distribution for continuous variables was assessed by the Shapiro‒Wilk test. Continuous variables were analyzed for differences between groups using Student’s *t* test when normally distributed or via the Mann–Whitney *U* test when nonnormally distributed. Survival curves were calculated using the Kaplan–Meier method. Cox proportional hazard model analysis was used to estimate the cumulative risk (hazard ratio, HR with 95% confidence interval) of initiating therapy with TKIs during follow-up. Receiver-operating characteristic (ROC) analyses were performed to identify the cut-off value for the time from the diagnosis of radioiodine-refractory disease to disease progression that was able to predict the indication for TKIs therapy with the best sensitivity (SN) and specificity (SP) according to the Youden index. The level of statistical significance was set for *p* less than or equal to 0.05. All statistical analyses were performed using SPSS (IBM SPSS Statistics, version 25).

## Results

### Clinical and histological characteristics at the diagnosis of the patients

Among the study group, 99/279 patients were addressed to TKIs’ treatment (Group A), while 180/279 (Group B) were maintained under active surveillance. The mean follow-up of Group A (from the initial diagnosis to the date of starting TKIs’ therapy) was similar to that of Group B (from the initial diagnosis to the last visit or death) [9.9 years (range 0.23–36.4 years) and 9.0 years (range 1.2–31.4 years), respectively].

As shown in Table 1, the comparison of the epidemiological and pathological features of the two groups demonstrated that, at the time of diagnosis, the patients of Group A were significantly older, had a larger primary tumor size, had a more frequent macroscopic extrathyroidal extension (ETE), and were characterized by a higher proportion of FTC, HCTC and PDTC.

**Table 1:** Demographic and pathological features of 99 patients with radioiodine-refractory thyroid cancer (RAIR-TC) addressed to tyrosine kinase therapy (Group A) and 180 controls with RAIR-TC under active surveillance (Group B)

	Group A (*N* = 99)	Group B (*N* = 180)	*p*
Mean age at diagnosis ± SD	57.1 ± 12.1	47.2 ± 17.8	< 0.001
Female gender (%)	47 (47.5)	95 (52.8)	> 0.05
Mean tumor size ± SD (cm)	4.1 ± 2.2	2.8 ± 1.8	< 0.001
Multifocality, *N* (%)^a^	50/84 (59.5)	81/148 (54.7)	> 0.05
Bilaterality, *N* (%)^a^	25/81 (30.9)	53/138 (38.4)	> 0.05
Macroscopic extrathyroidal invasion, *N* (%)^a^	17/89 (19.1)	13/166 (7.8)	0.027
Microscopic extrathyroidal invasion, *N* (%)^a^	47/89 (52.8)	104/166 (62.7)	> 0.05
Vascular invasion, *N* (%)^a^	45/86 (52.3)	62/148 (41.9)	> 0.05
Thyroiditis, *N* (%)^a^	14/84 (16.7)	26/149 (17.4)	> 0.05
PTC, *N* (%)^a^	56/98 (57.1)	142/179 (79.3)	0.001
FTC, *N* (%)^a^	23/98 (23.5)	17/179 (9.5)	
HCTC, *N* (%)^a^	9/98 (9.2)	8/179 (4.5)	
PDTC, *N* (%)^a^	10/98 (10.2)	12/179 (6.7)	
Aggressive variants, *N* (%)^a^	40/68 (58.8)	60/127 (47.2)	> 0.05
T1^a^	10/78 (12.8)	49/142 (34.5)	< 0.001
T2^a^	18/78 (23.1)	44/142 (30.1)	
T3^a^	33/78 (42.3)	39/142 (27.5)	
T4^a^	17/78 (21.8)	10/142 (7.0)	
Neck lymph-node metastasis, *N* (%)	44/95 (46.3)	108/175 (61.2)	0.015
DM, *N* (%)	34/95 (35.8)	25/175 (14.3)	< 0.001
Number of sites involved with DM, *N* (%)			
1 site only	27/95 (28.4)	23/175 (13.1)	< 0.001
2 sites or more	7/95 (7.4)	2/175 (1.1)	
Lung metastases, *N* (%)^a^	25/95 (26.3)	18/175 (10.3)	0.002
Bone metastases, *N* (%)^a^	10/95 (10.5)	8/175 (4.6)	> 0.05
ATA risk stratification, *N* (%)^a^			
Low	3/89 (3.4)	6/169 (3.6)	< 0.001
Intermediate	19/89 (21.3)	97/169 (57.4)	
High	67/89 (75.3)	68/169 (40.2)	
AJCC, *N* (%)^a^			
Stage 1	34/92 (37)	111/170 (65.3)	< 0.001
Stage 2	26/92 (28.3)	44/170 (25.9)	
Stage 3	6/92 (6.5)	3/170 (1.8)	
Stage 4a	1/92 (1.1)	0/170	
Stage 4b	25/92 (27.2)	12/170 (7.1)	

*AJCC* American Joint Cancer Committee, *ATA* American Thyroid Association, *DM* distant metastases, *FTC* follicular thyroid carcinoma, *HCTC* Hürthle cell thyroid carcinoma, *PDTC* poorly differentiated thyroid carcinoma, *PTC* papillary thyroid carcinoma, *SD* standard deviation

^a^Data regarding this pathological feature are missing for some patients

Concerning the extent of the disease at diagnosis (Table 1), Group A had their primary tumors more frequently classified as T3 and T4, and had a higher prevalence of distant metastases, mainly lung and bone metastases, but a lower prevalence of neck lymph-node metastases. Accordingly, the AJCC staging was significantly different between the two groups: Group A patients were diagnosed more frequently at a higher stage than Group B. Regarding the ATA risk of recurrence, Group A patients were more frequently stratified as high risk.

### Clinical and pathological characteristics at the time of RAIR diagnosis

The cumulative median activity of 131-I administered to Group A was 316 mCi (range 30–1718 mCi) and 272 mCi (range 30–1364 mCi) in Group B, and no significant difference was observed between the two groups when they were compared for this parameter (*p* = 0.133).

The median time from the initial diagnosis to RAIR diagnosis was similar in the two groups: 3.16 years (range 0.08–28.67 years) for Group A and 2.58 years (range 0.11–23.69 years) for Group B (*p* = 0.355). In Table 2, the distribution of the different categories of RAIR according to the ATA definition is reported. It is worth to note that patients who showed the absence of radioiodine uptake from the beginning (category 1) are equally distributed in the two groups of treated and not-treated patients. On the other hand, patients belonging to category 3 and 4 who were at least initially able to take up radioiodine were significantly more frequent in group A. As shown in Table 2, both the structural disease localization (i.e., local disease, lymph node, and distant metastases) and the sites of distant metastases at the time of RAIR diagnosis were significantly and differently distributed in the two groups. In particular, patients with local disease and or lymph-node metastases (both N1a and N1b were considered) were less frequent in group A in which, as expected, distant metastases were more represented.

**Table 2:** Clinical features at the time of diagnosis of radioiodine refractoriness (RAIR) in 99 patients with radioiodine-refractory thyroid cancer (RAIR-TC) addressed to tyrosine kinase therapy (Group A) and 180 controls with RAIR-TC under active surveillance (Group B)

	Group A (*N* = 99)	Group B (*N* = 180)	*p*
Category of RAIR^a^, *N* (%)			
1	50 (50.5)	89 (49.4)	
2	18 (18.2)	61 (33.9)	0.047
3	19 (19.2)	25 (13.9)	
4	12 (12.1)	5 (2.8)	
Disease localization at the time of the diagnosis of RAIR, *N* (%)			
Local disease and lymph-node metastases	44 (44.4)		
Lymph-node metastasis only	12 (12.1)		
DM only	55 (55.5)		
Sites of DM at the time of diagnosis of RAIR, *N* (%)			
Lung	74 (74.7)	70 (38.9)	< 0.001
Pleura	4 (4.0)	1 (0.6)	0.038
Bone	20 (20.2)	16 (8.9)	0.008
Brain	5 (5.0)	0	0.005
Liver	7 (7.1)	2 (1.1)	0.008

*DM* distant metastases

^a^Categories of RAIR: 1. the malignant/metastatic tissue does not ever concentrate radioiodine (RAI) (no uptake outside the thyroid bed at the first therapeutic whole-body scan); 2. the tumor tissue loses the ability to concentrate RAI after previous evidence of RAI-avid disease; 3. RAI is concentrated in some lesions but not in others; 4. DM progresses within 12–18 months from the last RAI treatment despite significant concentration of RAI

### Disease progression assessment

After the RAIR diagnosis, 93/99 (93.9%) patients in Group A were submitted to RECIST restaging of neoplastic disease, while 6 (6.1%) were immediately treated with TKIs, without restaging, since they were affected by a large tumor burden requiring immediate systemic therapy. All patients in Group B were restaged. The “restaged” 93 patients in Group A had at least one PD according to RECIST, and TKIs’ treatment was started after a median time of 7.2 years (range 0.5–30.7 years) from the initial diagnosis and after a median time of 2.5 years (range 0.1–19.9 years) from the RAIR diagnosis.

In Group B, the median time from diagnosis to the end of follow-up (last visit or death) was 9 years (range 1.2–31.4 years), while the median time from RAIR diagnosis to the end of follow-up was 4.8 years (range 0.53–24.7 years). In Group B, 103/180 (57.2%) never showed PD according to RECIST, while 77 patients (42.8%) presented PD (from 1 to 5). These latter patients were not submitted to TKIs due to the development of one or a few new or increasing lesions that were possible to treat with surgery or other local treatments in 44 (57.1%) cases (see Table 3 for details on their local treatments), to an increase in some asymptomatic small metastases that were kept under follow-up in 17 cases (22.1%), and to the development of new small lesions (< 1 cm) with no clinical significance in 16 cases (20.8%).

**Table 3:** Local treatments used in 44 patients who presented from 1 to 5 progressions of disease but were not treated with tyrosine kinase therapy. A total of 74 treatments alone or combined were used

Type of local treatment	Number of local treatments performed (%)
Surgical treatment of local recurrence/lymphadenectomy	31/74 (42)
External beam radiation therapy on the neck	19/74 (25.7)
External beam radiation therapy on the bone	9/74 (12.2)
Radiofrequency thermal ablation of local disease/lymph-node metastases	4/74 (5.4)
Surgical excision of bone metastases	2/74 (2.7)
Endoscopic laser unblocking and tracheal recanalization	2/74 (2.7)
Surgical excision of lung metastases	2/74 (2.7)
Transarterial chemoembolization or radioembolization of liver metastases	2/74 (2.7)
External beam radiation therapy on lung metastases	1/74 (1.3)
Surgical excision of pancreatic metastasis	1/74 (1.3)
Stereotaxic radiosurgery on brain lesions	1/74 (1.3)

The median time from RAIR diagnosis to the first PD was significantly shorter in Group A than in Group B [Group A median time 1.37 years (range 0.01–11.33 years), Group B median time: 2.9 years (range 0.75–16.4 years); *p* < 0.001]. A ROC curve analysis identified a cutoff of 25 months from RAIR diagnosis to the first PD that was associated with the indication to start systemic therapy [area under the curve (AUC) 0.713, *p* < 0.001] (sensitivity 69.8% and specificity 64.9%).

**Fig. 1:**
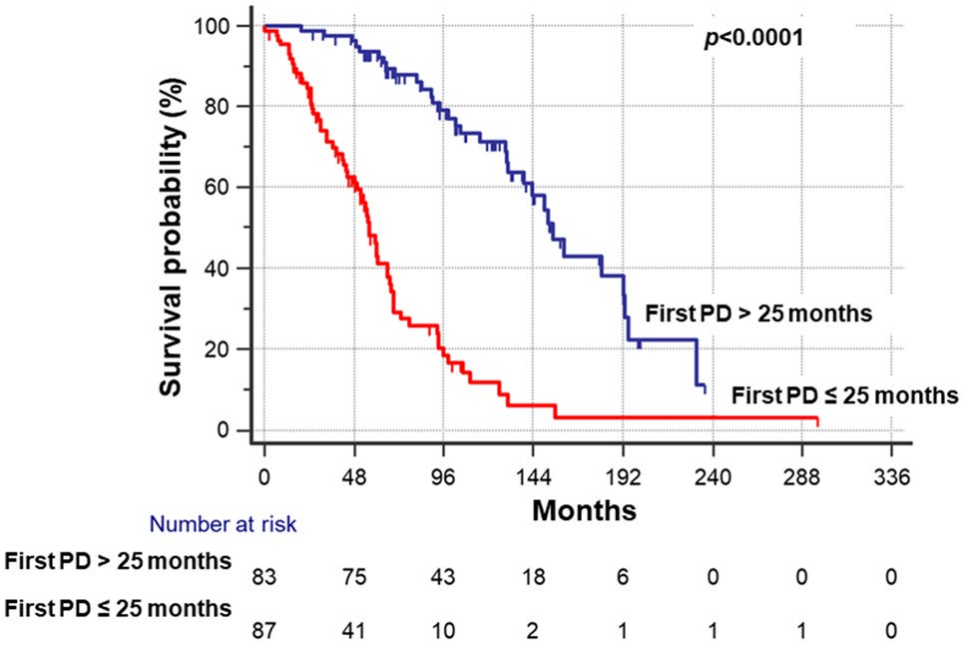
Cumulative probability of starting TKIs’ therapy considering the first progression of disease (PD) occurring within 25 months from the diagnosis of radioiodine refractoriness (cut-off time = 25 months was identified by ROC curve analysis; AUC: 0.713, *p* < 0.001)

Using this cutoff, we divided the patients with PD (*n* = 170, including 93 patients of group A and 77 patients of group B) into two subsets: subjects who had the first PD within 25 months from the RAIR diagnosis (*n* = 87) and those who were diagnosed with a first PD over 25 months from the RAIR diagnosis (*n* = 83). Patients in the first subset had a 6.5-fold higher risk to be addressed to TKIs therapy than patients in the second subset (hazard ratio HR 6.58; 95% CI 4.14–10.47;* p* < 0.0001) (Fig. 1). To note that six patients of group A were excluded from this analysis, because they were immediately treated with TKIs, without restaging, since they were affected by a large tumor burden requiring immediate systemic therapy.

## Discussion

The greatest challenge in treating RAIR-TC patients is the identification of the most appropriate time to start systemic therapy, particularly TKIs. This issue is mainly related to the considerable percentage of patients treated with TKIs that experience some adverse events that could severely worsen their quality of life [14–17]. So far, a very similar follow-up strategy is applied to all RAIR-TC patients thus exposing some of them to an excessive number of clinical examinations and imaging.

In the phase 3 DECISION, SELECT, and COSMI-311 studies, which led to the approval of the currently available TKIs, sorafenib, lenvatinib, and cabozantinib, the most important inclusion criterion to enroll a patient was represented by the radiological demonstration of PD, in a given time interval and established according to RECIST [6–8]. Once the three drugs were approved for commercial use, although the same criterion regarding the demonstration of PD has been extended to clinical practice in the real world, other clinical parameters were introduced for the identification of the right candidates for TKIs’ treatment. In particular, the clinical judgment of the impact of the tumor burden (either too large or too small), independent of PD, is currently used in real life.

In the present study, we have reported the experience of a single university center in the selection of RAIR-TC patients who need to be treated with TKIs. We distinguished two groups of patients, one that was treated with TKIs drugs (Group A) and one that was not treated (Group B), at least until the end of our observation period. These two groups showed several statistically significant epidemiological and pathological differences, thus demonstrating that, in clinical practice, there are two different groups of RAIR-TC patients. It is worth to note that while the time elapsed from the initial diagnosis to the definition of RAIR was similar, the time elapsed from the RAIR definition to PD was significantly shorter in the group that was treated, thus indicating that there are some cases that are rapidly progressing and others that are slower. The rate of growth can vary significantly in thyroid cancer, and in many cases, it is rather slow [10]. This parameter must be taken into consideration when deciding to start TKIs’ therapy.

One could argue that the beginning of TKIs’ treatment in the second group could be just a matter of time, but we observed that the median time of follow-up of Group A (from diagnosis to TKIs start) and Group B (from diagnosis to last visit or death) was similar in the two groups (7.2 years and 9 years, respectively), and thus, Group B had the same temporal probability of initiating the therapy, but evidently, the neoplastic growth rate was slower in this group, as previously supposed.

Moreover, even in cases of Group B with evident PD according to RECIST, the progression was minimal or due to the onset of small lesions, which, according not only to clinical judgment but also following the suggestions of the experts [18], did not justify the need to start TKIs’ therapy. One of the reasons to not start TKIs’ therapy in these cases is the awareness that adverse events due to these drugs will have a significant impact on the quality of life of patients [18, 19]. This limitation could be overcome once the high specificity for a mutated oncogene of the new generation of TKIs [20, 21] will significantly reduce the toxicity due to the “off-targeted” activities of the multikinase inhibitors we are currently using in clinical practice. Based on this better tolerability, in the near future, selecting the beginning of systemic treatment may no longer be a challenge in RAIR-TC management.

According to our study, several parameters already present at the time of diagnosis can be predictors of a more pressing necessity to start TKI therapy. At the initial diagnosis, Group A patients were significantly older and had a larger primary tumor, a more frequent ETE, a more aggressive histotype, and a greater extension of the primary tumor than Group B patients. Group A also showed a higher prevalence of distant metastases at diagnosis and a higher risk of recurrence, defined according to the ATA guidelines [12]. All of these features are already well-known predictive factors of a poor prognosis and a worse outcome, both in terms of persistence/recurrence of structural disease and mortality [2, 22–26]. This study confirmed that TCs with these “aggressive features” at initial diagnosis have a more aggressive behavior and are characterized by a more rapid growth rate, and for these reasons, they have a higher probability of requiring systemic therapy within a short period after the RAIR occurs. These patients must, therefore, be monitored more carefully from the beginning and rather frequently (i.e., every 4–6 months) to seize the most appropriate moment to start systemic therapy. Furthermore, since precision medicine is more available these days [27], these cases should be subjected to a complete genetic analysis right away, as they will almost certainly need to start systemic therapy and knowing their genetic profile will help with choosing the most appropriate therapy in a timely fashion.

A data point that deserves to be underlined is that at RAIR diagnosis, a disease limited to neck lymph nodes was detected in 12.1% of Group A, and in 57.2% of Group B, with a significant difference between the two groups. Therefore, metastatic disease confined to lymph nodes at RAIR diagnosis, although refractory to radioiodine treatment, can be considered to have a prognostic role of less or no need to start therapy. These data are in line with the concept that lymph-node metastases alone, in general, have indolent behavior, and this is mainly due to the slow growth of this kind of secondary lesion [28].

Regarding the PD and the tumor burden, this study showed that most patients who required TKIs (93.9%) had at least one PD according to RECIST, while in only 6.1% of patients, the tumor burden was so significant to require an immediate TKIs’ start. PD according to RECIST was also identified in Group B, and 42.7% of patients had several progressions (from 1 to 5) according to RECIST. Analyzing this subgroup of patients, they did not require systemic therapy, because in most cases, the PD was of a single lesion or organ, and it was possible to approach the progressive lesions with local treatments, while in other cases, the tumor burden and the progression were considered not clinically significant. Thus, within the therapeutic algorithm, it is important to evaluate the site, number, and size of metastatic lesions, as they may determine the feasibility of the surgical approach or other local treatments [29, 30]. In the present study, local treatments showed efficacy in avoiding systemic treatment with TKIs in approximately 57% of patients in Group B despite the progression of the disease. These data show the importance of the evaluation of all possible therapeutic strategies before proceeding with TKIs treatment, as recommended by the guidelines [12, 31].

We also demonstrated that patients who had their first PD according to RECIST within 25 months of RAIR diagnosis had a 6.5-fold higher risk of initiating systemic therapy with TKI. Therefore, we hypothesize that a closer clinical and radiological follow-up might be recommended in the first 2 years after RAIR diagnosis. According to our data, we can say that if a patient shows no progression within this period, it is likely that they will have a slowly progressive disease that will not need TKIs, and a less stringent follow-up could be proposed thereafter. Although we should keep in mind that a low sensitivity and specificity were found for this “cut off” time, additional studies are needed to validate it and to investigate its impact on endpoints such as mortality and PFS.

In conclusion, among patients with RAIR-TC, those with a primary tumor with aggressive pathological characteristics, more advanced disease at diagnosis, and the first PD occurring within 25 months of RAIR diagnosis were more frequently addressed to TKIs’ therapy. According to our results, a more tailored follow-up can be applied to RAIR-TC patients. A too strict monitoring and too many imaging evaluations could be avoided in those with less aggressive features and low rate of progression. Conversely, RAIR-TC with an advanced stage at diagnosis and a first PD occurring within 25 months from RAIR diagnosis will require a more stringent follow-up to avoid a late start of TKIs. Moreover, this group could benefit from an immediate molecular analysis to choose the appropriate targeted drug. To date, our work appears to be the only monocentric study with a considerable number of patients with RAIR-TC, aiming to describe the clinical and pathological characteristics of patients requiring systemic therapy with TKIs and identifying a tailored timing to follow-up these patients.

## Data Availability

Not applicable.
